# Survey of Candidate Single-Nucleotide Polymorphisms in SLC11A1, TLR4, NOD2, PGLYRP1, and IFNγ in Ankole Longhorn Cattle in Central Region of Uganda to Determine Their Role in *Mycobacterium avium* Subspecies *paratuberculosis* Infection Outcome

**DOI:** 10.3389/fvets.2021.614518

**Published:** 2021-02-12

**Authors:** Julius Boniface Okuni, Mathias Afayoa, Lonzy Ojok

**Affiliations:** ^1^Department of Pharmacy, Veterinary Clinical and Companion Animal Medicine, College of Veterinary Medicine, Animal Resources and Biosecurity (COVAB), Makerere University, Kampala, Uganda; ^2^Department of Pathology, Faculty of Medicine, Gulu University, Gulu, Uganda

**Keywords:** *Mycobacterium avium* subspecies *paratuberculosis*, Ankole cattle, SNPs, SLC11A1, TLR4, Nod2, IFNγ, PGLYRP1

## Abstract

*Mycobacterium avium* ssp. *paratuberculosis* (MAP) is the cause of Johne's disease (JD) in a wide range of domestic and wild ruminants. Single-nucleotide polymorphisms (SNPs) in several genes including solute-like carrier 11A1 (SLC11A1), interferon gamma (IFNγ), Toll-like receptor 4 (TLR4), nucleotide-binding oligomerization domain 2 gene (NOD2), and bovine peptidoglycan recognition protein 1 (PGLYRP1) have been implicated in influencing the infection outcome of MAP in cattle. We have carried out a survey in a population of Ankole cattle from three districts in the central region of Uganda including Isingiro, Lyantonde, and Rakai to determine the role played by several SNPs on the above genes in the infection outcome of local cattle in Uganda. Nine hundred fifty-five heads of cattle obtained from 93 herds were tested using ELISA. Thirty-five ELISA-positive cattle and 35 negative herd mates from a total of 955 cattle tested for MAP were genotyped using iPLEX MassARRAY genotyping systems to detect the presence of a total of 13 SNPS in five different genes (SLC11A1, IFNγ, TLR4, NOD2, and PGLYRP1). The cow-level prevalence of MAP infection in Ankole Longhorn cattle in the three districts was 3.98% (35/955), while the herd-level prevalence was 27.9% and within-herd prevalence was 12 ± 1.5% (95% CI = 9.1–14.8%). The genotypes and allele frequencies of the MAP-positive cattle were compared with those of their ELISA-negative herd mates to determine the significance of the polymorphisms. The results showed that SNPs rs109915208, rs110514940, and rs110905610 on SLC11A1, c.480G>A and c.625C>A on PGLYRP1, and c.2021C>T on TLR4 were monomorphic in both seropositive and seronegative cattle and therefore had no influence on the infection outcome. The remaining SNPs studied in the five genes [*SLC11A1*: rs109614179; *TLR4*: rs29017188 (c.226G>C), c.2021C>T; NOD2: rs110536091, rs111009394; *PGLYRP1*: c.102G>C, c.480G>A, c.625C>A; *IFN*γ: rs110853455] were polymorphic, but their allele and genotype frequencies did not show any significant difference between the seropositive and seronegative cattle. No significant difference was observed for any haplotype at the gene level.

## Introduction

*Mycobacterium avium* ssp. *paratuberculosis* (MAP) is the cause of paratuberculosis (PTB) or Johne's disease (JD), a chronic granulomatous enteritis characterized by projectile watery diarrhea in cattle (or loose feces in other species), marked weight loss, submandibular edema, and eventual death (1). PTB causes serious economic loses to the dairy industry in developed countries (2), and it is becoming increasingly relevant in Africa as well (3). Economic impacts of this disease stem from poor growth rate, progressive weight loss, reduced milk production, increased culling, death in affected animals, and loss of market for replacement heifers from affected farms (4).

The biggest risk factor for any herd or population of susceptible cattle is the presence of a clinically affected animal that is shedding the causative agent. In such herds, infected cattle shed MAP through colostrum, milk, and feces and are ingested through feeding of milk or contaminated pasture and water (5). For decades, control of PTB has relied on testing and culling of infected livestock, purchase of stock from JD-free herds, and addition of lime on pasture to render the environment unfavorable for survival of MAP (6). Unfortunately, there has been limited success in these measures due to various difficulties such as lack of a highly sensitive test for the disease during the early stages of infection (7). Diagnosis of the disease relies on antibody detection and demonstration of MAP in feces, which is only possible during the late stages. The other challenge is that culling is extremely expensive and could practically shut down some farms with no guarantee that the MAP-free stock to be introduced will not get infected, since it takes a long time to get rid of MAP from the farm environment (8).

Recently, efforts toward control of MAP infection has turned to the fact that some breeds of cattle (9) and deer (10) appear to be more resistant, while others are more susceptible. MAP infection does not necessarily lead to PTB, since it has been observed that certain breeds and lineages within specific breeds appear to be more resistant, while others are more susceptible. There are noted variations in the severity and spectrum of lesions even among infected cattle (9). This could be attributed to differences in strains of MAP or the genotypes of the host species (11). Indeed, a number of genetic association studies have linked single-nucleotide polymorphisms (SNPs) in some genes to increased susceptibility or resistance to MAP infection.

So far, studies on the effect of SNPs on the most significant genes that play key roles in innate immunity, including Toll-like receptors (TLRs) 1, 2, 4, and 9; nucleotide-binding oligomerization domain 2 (NOD2), solute-like carrier 11A1 (SLC11A1), interferon gamma (IFNγ), and peptidoglycan recognition protein 1 (PGLYRP1), have been done on different breeds of cattle across the world mostly on *Bos taurus* breeds of cattle (12–18), but there is scarcely any report on the frequency of those polymorphisms and their influence on African cattle. The only study of *Bos indicus* but outside Africa is that of Sadana et al. (19), who investigated the association of MAP infection status with SNPs on SLC11A1, TLR2, NOD2, and IFNγ in Indian cattle.

African breeds of cattle have been recognized for their innate resistance to some of the most important diseases of cattle such as tickborne infections and nagana (20). In Uganda, two indigenous breeds, Ankole and zebu cattle, are the backbone of both milk and meat production in the country. These breeds are known for their natural resistance to many diseases (20, 21), but no investigation has been done to understand how they would respond to MAP infection. Ankole Longhorn cattle is a breed of *B. indicus* cattle that has a characteristic long horn phenotype and is one of the two indigenous breeds in Uganda (21). Two published surveys have indicated that MAP infections in Ankole cattle and their crossbreeds have similar rates of infection as exotic breeds of cattle (22, 23). This finding is of great concern because, at the moment, there is no control measure against PTB in Uganda as is the case in most African countries. However, the studies were carried out in mixed breed herds, and therefore, there was a likelihood that the disease incidences in Ankole cattle that tested positive could have been influenced by the herd characteristics such as high stocking density, presence of large number of very susceptible cattle, and husbandry practices. A report (24) noted earlier that there is a wide lesion spectrum in cattle that are slaughtered at an abattoir in Kampala, which suggests differences in either susceptibility, resistance of the cattle, or virulence of strains of infecting MAP organism or the stage of infection. This study has been carried out to determine allele frequency and the effect of previously studied SNPs in SLCA11A1, NOD5, TLR-4, IFNγ, and PGLYRP1 on serostatus of Ankole cattle to MAP infection.

## Materials and Methods

### Study Area

**Figure 1:**
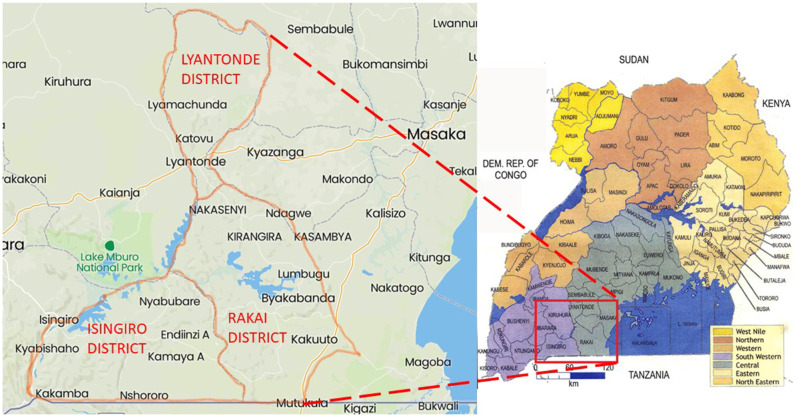
Map showing the location of Isingiro, Lyantonde, and Rakai districts.

This study was carried out in three districts of south-central Uganda, namely, Isingiro, Lyantonde, and Rakai, which lie on the southern end of a semiarid belt known as the cattle corridor, which runs across Uganda from the south-south-west to the north-east and was historically well-known for pastoralism. The area lies between the southwestern shore of Lake Victoria known as Sango Bay to the East and the Uganda–Tanzania Border to the south in Rakai, to Nakivale in Isingiro, extending northward toward Lake Mburo and Kyotera. The area has one long dry spell and two short rainy seasons. The vegetation cover consists of wooded semitropical grassland with undulating hills and valleys (Figure 1).

### Study Population and Sample Collection

The study population consisted of Ankole Longhorn cattle populations in the three districts. Care was taken to include only cattle from farms and areas with only pure Ankole cattle in herds without mixed breeds. Consent was requested and obtained from the livestock owners to enroll and sample their cattle. Baseline information such as herd size, husbandry practices, history of clinical signs of PTB, and knowledge about the disease was obtained from each of the farmers who agreed to participate. A total of 93 herds that are kept under the traditional system of husbandry were sampled in the study, from which 955 samples were collected. Age of the cattle was obtained from the farmer and verified by checking the tooth eruption and wear. Age was recorded, and only cattle that was 2 years above were included in the study. Blood was obtained aseptically from the animals through the caudal or jugular vein following proper restraint. Two blood samples were obtained from each cow into a vacutainer: ~2 ml of blood into a heparinized tube and another 4 ml of blood into a plain vacutainer for serum extraction. The samples were both placed in a cool box and transported to the laboratory. Serum was extracted from the clotted blood collected in the plain vacutainers and was later tested for MAP.

### Serological Testing for *M. avium* ssp. *paratuberculosis* Antibodies

ELISA was performed on sera to test for MAP antibodies using a commercial ELISA kit from IDVET (Montpellier, France) according to the manufacturer's instruction. Optical density readings of the plates were taken at 450 nm using SpectroStar Nano ELISA reader (BMG LABTECH GmbH). To determine the serostatus, a sample to positive ratio was calculated using the formula: S/P = (Sample OD-NC-OD)/(PC-OD – NC-OD) × 100. Where S/P is the sample to positive ratio, Sample OD is the optical density reading for each sample, NC-OD is the mean optical density reading for the negative controls, and PC-OD is the mean optical density reading of the positive controls. A sample was considered positive if its S/P ratio is equal to or >70%.

### Sources of Single-Nucleotide Polymorphisms Studied

The SNPs reported in this paper were discovered by other authors and have been referenced in GenBank and ENSEMBL databases. For SLC11A1 gene, the SNPs included rs109614179, rs109915208, rs110905610, and rs110514940 (19). For NOD2 gene, the investigated SNPs are rs110536091 and rs111009394 (19), while for IFNγ, SNP rs110853455 (19) was investigated. The others were c.102G>C (ss104807451), c.480G>A (ss104807452), and c.625C>A (ss104807453) on the PGLYRP1 gene (17) and rs29017188, rs8193060, and c.2021C>T on the TLR4 gene (25, 26).

### Genotyping

A total of 35 (*n* = 955) ELISA-positive Ankole cattle from 26 farms across the three districts and 35 matched controls (Supplementary Table 1) were genotyped at different gene loci to determine the presence or absence of SNPs previously associated with MAP infection. DNA was extracted using Quick-DNA™ Microprep Kit (Zymo Research Corp., Freiburg, Germany) according to the manufacturer's instructions. DNA was diluted to a final concentration of 25 ng/μl and stored at −20°C. The flanking sequences of the above SNPs were retrieved, and locus-specific and extension primers targeting each of the 13 SNPS across the five genes were designed using Agena Assay Design software and synthesized unmodified under standard purification (Table 1). Primers were synthesized by Inqaba Biotec Laboratories (Pretoria, SA). Genotyping was done using iPLEX MassARRAY platform, which is based on matrix-assisted laser desorption ionization-time of flight (MALDI-TOF) mass spectrometry (Agena Biosciences). The reagents (iPLEX Pro Genotyping reagents) used were custom made for use in Agena MassRRAY system. Briefly, equimolar concentrations of the amplification primers were pooled into a working primer solution containing 0.5 mM of each primer. A PCR mix consisting of 1.8 μl of high-performance liquid chromatography (HPLC)-grade water, 0.5 μl of 10 × PCR buffer (20 mM MgCl_2_), 0.4 μl of MgCl_2_ (25 mM), 0.1 μl of dNTPs (25 mM each), 1 μl of primers (0.5 mM each), and 0.2 μl of polymerase (5 U/μl) was prepared in a total volume of 5 μl. The reaction conditions consisted of initial denaturation at 94°C for 2 min and 45 cycles at 94°C for 30 s, 56°C for 30 s, 72°C for 60 s in each cycle, and a final extension of 72°C for 5 min and cooled to 4°C. After the PCR reaction, 2 μl of shrimp alkaline phosphatase was added to each well and the mix was incubated at 37°C for 40 min and then at 80°C for 5 min. Single base extension of the reaction was carried out by addition of 2 μl of iPLEX-SBE master mix followed by PCR cycle of 94°C for 30 s, 95°C for 5 s, 52°C for 3 s, and 80°C for 3 s repeated five times. The whole amplification was repeated 60 times then a final extension of 72°C for 3 min and held at 15°C. All PCR amplifications were done using Mastercycler® Nexus machines (Eppendorf). The products were purified using resin and then spotted into a 384-well microchip plate using a nanodispenser (MassARRAY System with CPM 384). The chip was then read by the MALDI-TOF Mass Spectrometer (MassARRAY® Analyzer 4, Agena Biosciences), and data were obtained using Typer 4.1. software. The spectrographs of each call were visually checked to confirm the identity of the base calls. Only samples with coverage >87.5% were included in the final analysis.

**Table 1:** List of SNPS studied and the primers used.

**Gene**	**SNPs**	**Name of the oligo**	**Sequence of oligo**	**References**
Toll-like receptor 4 (TLR4)	rs29017188	TLR4aF	ACGTTGGATGGGTCTGCAGACGTTTTCTTC	(12)
		TLR4aR	ACGTTGGATGCTTAGCTTTCTGGACTTTCG	
		TLR4aE	CCTCTAACTTCCCCTC	
	rs8193060	TLR4bF	ACGTTGGATGTTATGAACCACTCCACTCGC	(27)
		TLR4bR	ACGTTGGATGTCCTTAGAGGCCATGATACG	
		TLR4bE	CCATGATACGGTTGAA	
	c.2021C>T	TLR4cF	ACGTTGGATGTTCCACCTGATGCTTCTTGC	
		TLR4cR	ACGTTGGATGGGCTCGAGTAGATGACAAAG	(27)
		TLR4cE	AGGGGCGAGAGCA	
Peptidoglycan recognition protein 1 (PGLYRP1)	c.102G>C	PGLYRP1aF	ACGTTGGATGTAGCGCACAGGCTGTCTTAG	(17)
		PGLYRP1aR	ACGTTGGATGTCAAGACTGCGGCAGCATC	
		PGLYRP1aE	GCACTTGGATGCCAGGGC	
	c.480G>A	PGLYRP1bF	ACGTTGGATGTTTATAGAGCTCGTCCCCTG	(17)
		PGLYRP1bR	ACGTTGGATGCGGGGATACCTGACTCCTAA	
		PGLYRP1bE	CGGTGTCCTTTGAC	
	c.625C>A	PGLYRP1cF	ACGTTGGATGATGGCAGGACACAGGAATAC	
		PGLYRP1cR	ACGTTGGATGACCCATCCCATCAGAAACCC	(17)
		PGLYRP1cE	AGAAACCCCACCGC	
Solute-like carrier 11A1 (SLC11A1)	rs109614179	SLC11A1bF	ACGTTGGATGCCAGATATGGCTCCATCTCC	
		SLC11A1bR	ACGTTGGATGTTCTCACTTAGGTAGGTCCC	(19)
		SLC11A1bE	GAGCCACAGCAAGC	
	rs109915208	SLC11A1cF	ACGTTGGATGTTCACTCACCCCACAGTACG	
		SLC11A1cR	ACGTTGGATGGAACAGGCCCTGAAGCAATG	(19)
		SLC11A1cE	GAAGCAATGCTCCCTGA	
	rs110905610	SLC11A1dF	ACGTTGGATGCAAGACCCCTCATTCCACTC	
		SLC11A1dR	ACGTTGGATGTGCGATGCTCATGAGGAATC	(19)
		SLC11AdE	CCACTCCCCTCCACAGGGT	
	rs110514940	SLC11A1eE	AAGGCCCACAGCTTCC	
Nucleotide-binding oligomerization domain 2 (NOD2)	rs110536091	CARD15aF		
		CARD15aR	ACGTTGGATGTCTCAATTTCCTCCGGAGAG	(19)
		CARD15aE	TCTCTTTGCTTTATCCC	
	rs111009394	CARD15bF	ACGTTGGATGACAACTCTGTGGGCGACATC	
		CARD15bR	ACGTTGGATGAGCATCACTCACTAAAGAGC	(19)
		CARD15bE	AAGAGCTTTGCAGAC	
Interferon gamma (IFNγ)	rs110853455	IFNγF	ACGTTGGATGCCAGCGCAAAGCCATAAATG	
		IFNγR	ACGTTGGATGGATTCTGACTTCTCTTCCGC	(19)
		IFNγE	TCTGAGGTTAGATTTTGG	

### Data Analysis

Data were analyzed using SHEsisPlus software (http://analysis.bio-x.cn/SHEsisMain.htm (28) to estimate the allele and genotype frequencies, pairwise linkage disequilibria, and odds ratio (OR) to compare the associations of alleles, genotypes, and haplotypes with MAP infection. Haplotypes were constructed using a threshold of 0.05 for lowest frequency. The *p*-values were corrected for multiple comparisons with Sidak correction, and a false discovery rate (FDR) was derived for each SNP.

### Ethical Approval

Ethical clearance for this study was granted by the Uganda National Council for Science and Technology (UNCST) under reference number A32ES. Sample collection was done according to the guidelines on the use of animals in research.

## Results

This study was done with the aim of determining the effect of selected SNPs on the seropositivity of Ankole Longhorn cattle to *M. avium* ssp. *paratuberculosis* in central Uganda. Ninety-three herds with only local Ankole cattle, ranging from 8 to 300 cattle and an average of 48 cattle were sampled. The mean age of the cows sampled was 5.95 ± 3.14 years. The herd-level prevalence was 27.9% (26/93), which is at least one cow infected within the herd. The cow-level prevalence was 3.98% and the within-herd prevalence rate ranged from 3 to 29% with a mean of 12 ± 1.5% (95% CI = 9.1–14.8%). The prevalence rates for the three districts were 4.9% for Rakai, 4.8% for Lyantonde, and 2.5% for Isingiro.

The husbandry practices among the farms consisted of communal grazing and shared water sources in communal valley dams, rivers, and streams. Few of the farmers owned private valley dams and had paddocked grazing land. Seventy-three percent (*n* = 93) of the farmers reported that they had experienced prolonged diarrheal episodes in their farms, and 30.4% of them reported that the diarrhea was non-responsive to treatment. There was considerable mobility for most of the herds during dry season and close sharing of both pasture and water sources. Approximately 73% (*n* = 93) of the farmers reported regular introduction of new stock into their herds, either as gifts from other cattle keepers, purchases, or bulls for breeding but lacked knowledge (89.2%) about PTB. Water logging on the farms was reported by 17.3%.

The results of the MassARRAY genotyping indicated that SNP c.2021G>T on the TLR4 gene, c.480G>A and c.625C>A on the PGLYRP1 gene, and rs109915208, rs110905610, and rs110514940 on the SLC11A1 gene were monomorphic in both cases and controls and thus had no effect on the infection outcome in the population. The remaining SNPs, namely, rs29017188 (c.226G>C) and rs8193060 (c.1656C>T) on the TLR4 gene, c.480G>A and c.625C>A on the PGLYRP1 gene, rs109614179 on the SLC11A1 gene, and rs110536091 and rs111009394 on the NOD2 gene and finally rs110853455 on the IFNγ gene, did not have any significant differences between seropositive and seronegative animals. The alternative alleles for each of these SNPs are shown in Table 2. The frequencies of alleles and genotypes of the different SNPs across five genes SLC11A1, CARD15, IFNγ, TLR4, and PGLYRP1 and their individual effects are shown in Tables 2, 3. All polymorphic SNPs were in Hardy–Weinberg equilibrium, except rs109614179. There was a strong linkage disequilibrium for SNPs on NOD2 (*D'* = 1) and TLR4 (*D'* = 0.98). Haplotype analysis of SNPs within the different genes (Table 4) showed that haplotype CGCCT was significantly more represented in cases compared to controls (*p* = 0.053, *OR* = 2.236). Another haplotype CACGT has a stronger *p*-value 0.048 but with an *OR* < 1. However, in both cases, the FDR is 0.116.

**Table 2:** Allele frequencies of the different SNPs in Ankole cattle with positive and negative serostatus for *Mycobacterium avium* ssp. *paratuberculosis*.

**Gene**	**SNPs**	**Frequency of minor and major alleles**			***p*-value**	**Corrected *p*-value (SSD)**	**FDR**
		**Alleles**	**MAP positive**	**MAP negative**			
Toll-like receptor 4 (TLR4)	rs29017188	C	63 (0.900)	66 (0.943)	0.346025	–	–
		G	7 (0.100)	4 (0.057)			
	rs8193060	C	28 (0.400	24 (0.343)	0.484160	0.929	0.968
		T	42 (0.600)	46 (0.657)			
	c.2021C>T	C	70 (1.000	69 (0.986)	0.315563	–	–
		T	0 (0.000)	1 (0.014)			
Peptidoglycan recognition protein 1 (PGLYRP1)	c.102G>C	C	55 (0.786	15 (0.214)	0.017254	0.099	0.103
		G	42 (0.600)	28 (0.400)			
	c.480G>A	G	70 (1.000)	70 (1.000)	–	–	–
		A	0	0			
	c.625C>A	C	70 (1.000)	70 (1.000)	–	–	–
		A	0	0			
Solute-like carrier 11A1	rs109614179	A	42 (0.600)	30 (0.455)	0.089418	0.373	–
		G	28 (0.400)	36 (0.545)			
	rs109915208	C	70 (1.000)	66(1.000	–	–	–
	rs110905610	C	70 (1.000)	66 (1.000)			
	rs110514940	C	70 (1.000)	66 (1.000)	–	–	–
Nucleotide-binding oligomerization domain 2 (NOD2)	rs110536091	C	47 (0.671)	42 (0.677)	0.941550	0.984	0.969
		T	23 (0.329)	20 (0.323)			
	rs111009394	C	47 (0.671)	45 (0.682)	0.959511	0.989	–
		T	23 (0.329)	21 (0.318)			
Interferon gamma (IFNγ)	rs110853455	A	21 (0.300)	20 (0.303)	0.969285	0.989	0.969
		G	49 (0.700)	46 (0.697)			

*p, Pearson's p-value; SSD, Sidak step-down adjusted p-values for strong control of the familywise Type I error rate (FWER); FDR, adjusted p-values for the (29) step-up false discovery rate controlling procedure*.

**Table 3:** Frequency of different genotypes among cattle with positive and negative serostatus for MAP.

**Gene**	**SNPs**	**Genotype frequency**			***p*-value**	**Corrected *p*-value SSD**	**FDR**
		**Genotype**	**MAP positive**	**MAP negative**			
Toll-like receptor 4 (TLR4)	rs29017188	C/C	28 (0.800)	31 (0.886)	0.324487	-	-
		C/G	7 (0.200)	4 (0.114)			
		G/G	0 (0)	0 (0)			
	rs8193060	C/C	6 (0.171)	4 (0.114)	0.762290	0.986	0.959
		C/T	16 (0.457)	16 (0.457)			
		T/T	13 (0.371)	15 (0.429)			
	c.2021C>T	C/C	35 (1.000)	34 (0.971)	–	-	-
		C/T	0 (0)	1 (0.029)			
		T/T	0 (0)	0 (0)			
Peptidoglycan recognition protein 1 (PGLYRP1)	c.102G>C	C/C	22 (0.629)	12 (0.343)	0.051908	0.233	0.155
		C/G	11 (0.314)	18 (0.514)			
		G/G	2 (0.057)	5 (0.143)			
	c.480G>A	G/G	35 (1.000)	35 (1.000)	–		-
	c.625C>A	C/C	35 (1.000)	35 (1.000)	–		-
Solute-like carrier 11A1	rs109614179	A/A	9 (0.257)	7 (0.212)	0.028287	0.158	0.155
		A/G	24 (0.686)	16 (0.485)			
		G/G	2 (0.057)	10 (0.303)			
	rs109915208	C/C	35 (1.000)	33 (1.000)	–		-
	rs110905610	G/G	35 (1.000)	33 (1.000)	–		-
	rs110514940	C/C	35 (1.000)	33 (1.000)	–		-
Nucleotide-binding oligomerization domain 2 (NOD2)	rs110536091	C/C	17 (0.486)	15 (0.484)	0.983237	0.997	0.959
		C/T	13 (0.371)	12 (0.387)			
		T/T	5 (0.143)	4 (0.129)			
	rs111009394	C/C	17 (0.486)	16 (0.485)	0.959511	0.997	0.959
		C/T	13 (0.371)	13 (0.394)			
		T/T	5 (0.143)	4 (0.121)			
Interferon gamma (IFNγ)	rs110853455	A/A	4 (0.114)	2 (0.061)	0.551026	0.959	0.959
		A/G	13 (0.371)	16 (0.485)			
		G/G	18 (0.514)	15 (0.455)			

*p, Pearson's p-value; SSD, Sidak step-down adjusted p-values for strong control of the familywise Type I error rate (FWER); FDR, adjusted p-values for the (29) step-up false discovery rate controlling procedure*.

**Table 4:** Haplotypes inferred using SNPs rs109614179, rs110536091, rs110853455, rs111009394, c.102G>C (PGLYRP1), and rs8193060.

**Haplotype**	**Case (freq)**	**Control (freq)**	**Chi2**	**Pearson's p**	**OR [95% CI]**	**SSD**	**FDR**
CGCGT	3 (0.042)	8 (0.129)	2.466	0.116	0.347	0.579	0.215
TATCT	4 (0.057)	3 (0.048)	0.15	0.698	1.353	0.966	0.756
CGCCT^*^	18 (0.257)	9 (0.145)	3.716	0.053	2.346	0.363	0.116
CGCCC	9 (0.128)	11 (0.177)	0.233	0.629	0.791	0.966	0.743
CACGT^*^	2 (0.028)	8 (0.129)	3.876	0.048	0.227	0.363	0.116
TATCC	8 (0.114)	6 (0.096)	0.317	0.573	1.376	0.966	0.743
CACCT	6 (0.085)	2 (0.032)	2.121	0.145	3.187	0.61	0.236
CGCGC	8 (0.114)	4 (0.064)	1.458	0.227	2.129	0.724	0.328
TGTCT	9 (0.128)	8 (0.129)	0.066	0.795	1.143	0.966	0.795

*FDR, false discovery rate; OR, odds ratio; Sidak SD (Sidak's correction of p-value)*.

* *Haplotype CGCCT is significantly more represented in cases compared to controls with an OR of 2.346. CACGT also has a significant p-value but a small OR < 1*.

## Discussion

PTB is an emerging disease in Uganda and, owing to the lack of awareness and control, may be considered a neglected disease. The findings indicate that the prevalence of MAP infection in Ankole cattle is within the same range as it has been reported in mixed breed study in other districts within the central region of Uganda. This has disproved the long-held view that MAP infection of Ankole Longhorn cattle is due to transmission from highly susceptible exotic cattle. In the current study, the prevalence of 3.98% is comparable to 3.7% earlier on reported in Wakiso, Mpigi, and Luwero in mixed breed study (22); 4.7% in slaughtered cattle (24) but significantly lower than reported in indigenous breeds in Wakiso (23). Overall, the cow-level prevalence of MAP infection in these districts is still low compared to some countries in the world like Canada (30) and Denmark (31), but the herd-level prevalence of 29.3 is considerably high and is a cause for worry if no control measures are instituted. The finding of MAP-infected cattle in an area with one of the most traditional systems of husbandry indicates that PTB is well-established in the local cattle population in Uganda. This is especially so because no single prevalence survey in Uganda has returned a zero-prevalence rate in any district. Practices such as communal grazing and watering, livestock movements in search of water, and pasture during the dry season, all of which were responded to in the affirmative by all the farmers mean that it will be very difficult to control the disease in this region.

The results of genotyping show that about half of the SNPs in this study are monomorphic in the Ugandan Ankole Longhorn cattle and therefore may not play any role in the resistance or susceptibility to MAP among Ankole cattle. The other SNPs that were polymorphic also did not show a significant difference between cases and control.

Pairwise and single-site analysis of the data from this study using SHESis Plus software (http://analysis.bio-x.cn/SHEsisMain.htm) with *p*-value correction and FDR has led us to conclude that there is no significant difference in the allele and genotype frequency between the cases and control. However, several other studies that had reported an association between some of the SNPs and serostatus (12, 14, 32) in different breeds did not report any *p*-value correction using Bonferroni, Holms, or Sidak methods, and none also reported FDR values, and yet in some instances, the *p*-values were also marginal. Based on this observation, we believe that some of our findings may be comparable to those in some of the published reports.

Although SNP c.102G>C on PGLYRP1 gene had an uncorrected *p*-value of 0.0172 (Table 2), when corrected for multiple comparisons, its *p*-value became 0.099 and the FDR was 0.103 and thus was not significant. Pant et al. (17) have investigated the linkage between SNP c.102G>C, c.480G>A, and c.625C>A on the PGLYRP1 gene and MAP infection status in Holstein Friesian cattle and reported that c.480G>A is associated with MAP infection. In our study, however, c.480G>A was monomorphic and allele A has zero frequency. At present, there is scarcely any other study on the role of SNPs of this gene with respect to MAP or PTB.

SLC11A1 gene (SLC11A1) codes for the natural resistance-associated macrophage protein (NRAMP1) (33) and some alleles of SLC11A1 have been shown to confer the resistance against MAP to BALB/C mice (34), to cattle against diseases such as tuberculosis (35, 36), brucellosis (37), salmonellosis (38), and conferment of udder immunity (39). In this study, four SNPs on this gene identified as rs109614179, rs109915208, rs110905610, and rs110514940 were investigated for their association. Three of these SNPs (rs109915208, rs110905610, and rs110514940) were monomorphic, indicating that either they are monomorphic in the whole Ankole cattle population or the minor allele has very low allele frequency while rs109614179 was not significantly different in cases and controls. Regarding the significance of the SNPs on SLC11A1, our findings are similar to that of Sadana et al. (19), who did not find any significant association between these SNPs and MAP infection in Indian cattle. Likewise, there was no association between MAP serostatus and SNPs in IFNγ and TLR4, which we studied. Although other studies have reported an association, for example (19), found between MAP infection status with only IFNγ SNP rs110853455 and not with SNPs on SLC11A1, NOD2/CARD15, which were the focus of our investigation, and Sharma et al. (18) noted that the haplotype C/T of the SNPs c.226C>T and c.2021C>T on TLR4 was associated with susceptibility to MAP infection in Holsteins, yet in our case, both of these SNPs were not related to MAP infection status. Similarly, although Pinedo et al. (13) found an association between SNP c.2197T>C on the NOD2 gene and MAP infection in Holsteins and Brahman-Angus of Florida and Küpper et al. (15) reported an association between SNP g.521G>A on the same gene in German Holstein cattle, those SNPs were different from the ones in our study and therefore lacked a close comparison. Just like other innate immunity genes, NOD2 is known for its role in natural host resistance to bacterial infection (40) and therefore warrants investigation of its polymorphisms in association with MAP.

We aimed at determining the effect of the SNPs in the five genes enumerated here because they have been studied in Indian cattle (19), since Ankole Longhorn cattle are also *B. indicus* and are therefore closer to the Indian breeds than the Holstein Friesian and other *B. taurus* breeds in other studies. While Sadana et al. (19) used PCR-RLFP for genotyping, we used iPLEX MassARRAY for genotyping except for SNP rs11053609, where we used both methods and obtained the same result, thus validating the use of MassARRAY genotyping. We note that although each of the SNPs had little or no significant effect, when considered together, some haplotypes, e.g., CGCCT (Table 4), may have significant association with the infection. This also may suggest that if the number of individuals in the study was bigger, in hundreds or thousands, it could have been possible to observe a clear association for some of the SNPs. Our sample size was small because the cases and controls were derived from a study aimed at determining the seroprevalence of MAP among pure Ankole cattle herds in the central region of the country. Overall, 955 heads of cattle were sampled, and 35 of them (3.6%) were seropositive. Due to the lack of well-characterized MAP-positive and MAP-negative herds or a database of JD-positive cattle in the country, it was difficult to come up with a large number of samples for this kind of study. It would have required a very large survey, which was beyond our resources, and furthermore, characterizing herds as MAP resistant would still be a challenge. There are also studies that have used small sample sizes due to the same reason and yet have reported interesting results (27). Given these limitations, we have used a method similar to that in other studies in which seronegative herd mates were considered controls (17, 25, 41), although we did not have the opportunity to confirm seronegative status by retesting. Based on our knowledge of this disease, these controls might be animals at preclinical stage or animals that may test positive on a second testing. We therefore believe that repeated studies especially with a larger number of animals will be necessary to concretely determine the significance of each of these SNPs in Ankole cattle with regard to MAP. Those SNPs that are monomorphic appear to be truly so or may represent SNPs whose minor alleles may be <5%, which would have been otherwise removed from the analysis, as this was the set limit for the minimum allele frequency during the analysis. It is not possible to draw comparisons between our findings and most of the previous studies, since most of the studies cited have used different SNPs, moreover, even with different nomenclature, and only one or a few SNPs being studied in each case. The studies cited point to the general indication that SLC11A1, TLR (1, 2, and 4), NOD2, PGLYRP1, and IFNγ have functional mutations that influence the outcome of cattle infection with MAP. More studies on the effect of the different SNPs on susceptibility or resistance within a breed are still necessary for future application for marker-assisted selection of individuals that are resistant and therefore achieve the control of PTB. To our knowledge, this is the first study done to assess the relevance of these SNPs in association with any infectious disease in Ankole cattle in Uganda and therefore provides key information regarding some of the SNPs, especially those that are now known to have limited allele diversity and those that may have significance for investigating the association with MAP. More studies are necessary to unravel their significance, more so using a larger sample size and well-characterized herds. Future studies may also aim at discovering more SNPs and other polymorphic loci, especially using genome-wide association studies.

In conclusion, the prevalence of MAP infection in Ankole Longhorn cattle in the districts of Isingiro, Lyantonde, and Rakai in central Uganda is still low like it is in the neighboring districts but indicates that the infection is equally established in the native cattle population under its traditional farming system. Furthermore, genotyping of SNPs previously reported to be associated with seropositivity to MAP is monomorphic in our study population, whereas the remaining are not significantly associated with cases compared with controls, and therefore, there is a need to carry out more studies using larger sample sizes to determine their population significance and genome-wide association studies that can lead to the discovery of other genetic markers that might be more relevant to any future prospect of marker-assisted selection in Ankole cattle.

## Data Availability Statement

The datasets presented in this study can be found online in the Supplementary Material. The details of the SNPs used and their accession numbers can be found in GenBank database.

## Ethics Statement

The animal study was reviewed and approved by Uganda National Council for Science and Technology (UNCST), Ministry of Science, Technology and ICT, Republic of Uganda (Ethical approval reference number: A32ES). Written informed consent was obtained from the owners for the participation of their animals in this study.

## Author Contributions

JO conceived the study and drafted the manuscript. JO, LO, and MA designed the study. JO and MA did the laboratory and fieldwork. LO and JO analyzed the data. All authors contributed to writing and approved the manuscript for publication.

## Conflict of Interest

The authors declare that the research was conducted in the absence of any commercial or financial relationships that could be construed as a potential conflict of interest.
